# Integrated, automated maintenance, expansion and differentiation of 2D and 3D patient-derived cellular models for high throughput drug screening

**DOI:** 10.1038/s41598-021-81129-3

**Published:** 2021-01-14

**Authors:** Ibrahim Boussaad, Gérald Cruciani, Silvia Bolognin, Paul Antony, Claire M. Dording, Yong-Jun Kwon, Peter Heutink, Eugenio Fava, Jens C. Schwamborn, Rejko Krüger

**Affiliations:** 1 Luxembourg Centre for Systems Biomedicine, Translational Neuroscience, University of Luxembourg, Luxembourg, Luxembourg; 2 Disease Modeling and Screening Platform (DMSP), Luxembourg Centre of Systems Biomedicine (Biomedicine), University of Luxembourg and Luxembourg Institute of Health (LIH), 6 Avenue du Swing, 4367 Belvaux, Luxembourg; 3 Developmental Biology, Luxembourg Centre for Systems Biomedicine, University of Luxembourg, Luxembourg, Luxembourg; 4 Oncology Department, Luxembourg Institute of Health (LIH), Strassen, Luxembourg; 5 German Center for Neurodegenerative Diseases (DZNE)-Tübingen &, Hertie Institute for Clinical Brain Research, Otfried Müller Strasse 23, 72076 Tübingen, Germany; 6 German Center for Neurodegenerative Diseases (DZNE) - Core Research Facilities and Services - Venusberg-Campus 1, Gebäude 99, 53127 Bonn, Germany; 7 Transversal Translational Medicine, Luxembourg Institute of Health (LIH), Strassen, Luxembourg; 8 Parkinson Research Clinic, Centre Hospitalier de Luxembourg (CHL), Luxembourg, Luxembourg

**Keywords:** High-throughput screening, Imaging, Phenotypic screening, Cellular neuroscience, Drug development

## Abstract

Patient-derived cellular models become an increasingly powerful tool to model human diseases for precision medicine approaches. The identification of robust cellular disease phenotypes in these models paved the way towards high throughput screenings (HTS) including the implementation of laboratory advanced automation. However, maintenance and expansion of cells for HTS remains largely manual work. Here, we describe an integrated, complex automated platform for HTS in a translational research setting also designed for maintenance and expansion of different cell types. The comprehensive design allows automation of all cultivation steps and is flexible for development of methods for variable cell types. We demonstrate protocols for controlled cell seeding, splitting and expansion of human fibroblasts, induced pluripotent stem cells (iPSC), and neural progenitor cells (NPC) that allow for subsequent differentiation into different cell types and image-based multiparametric screening. Furthermore, we provide automated protocols for neuronal differentiation of NPC in 2D culture and 3D midbrain organoids for HTS. The flexibility of this multitask platform makes it an ideal solution for translational research settings involving experiments on different patient-derived cellular models for precision medicine.

## Introduction

The two main approaches for drug discovery are target-based screenings, where the modulation of the activity of a previously identified druggable target is used as readout, and phenotypic screenings, in which the rescue of a phenotype in a disease model (cells, tissue, animal etc.) serves as readout^1,2^. In the past four decades, the majority of investment for drug discovery was biased towards target-based screenings^2^. These highly standardized large-scale assays can be performed in classical cell lines or even with isolated proteins in vitro. However, despite increasing investment, drug discovery has a high failure rate especially when it comes to neurodegenerative diseases (NDD)^3^. In fact, there are still no disease modifying drugs approved for the major NDD Alzheimer’s disease (AD) and Parkinson’s disease (PD)^3^. In the last 20 years only 5 first-in-class small molecule drugs for NDD were approved, of which none was discovered in target-based screenings but in phenotypic screenings^4^. Multifactorial diseases like PD are only poorly represented in a screening focusing on a single target molecule, as the “one-drug-one-target” approach does not take into account the complexity of such diseases^5^. In contrast, phenotypic screenings do not focus on a single target. Phenotypic screenings profited largely from the emergence and advancements of patient-based cellular models, i.e. induced pluripotent stem cell (iPSC) technologies. The development of differentiation protocols for a variety of disease-relevant cell types in xenofree conditions allows the generation of many new human cellular disease models suitable for drug screenings^6–9^. To model multifactorial diseases without any known molecular target iPSC can be derived from patient material, e.g. monogenic forms of NDD with clearly defined pathogenic mutations. Additionally, this also allows to model diseases affecting otherwise difficult to access cell types like neurons and produce them in quantities sufficient for a screening campaign. In this context, the importance of access to patient-derived data and models is increasing and becoming a game changer in drug discovery. From an industry perspective, it is crucial to find partners who can generate these patient-derived data and disease models. Here, academic institutions with their access to patient’s data and biospecimens and their expertise in disease modelling can step up to fill the gap of required screenable cellular disease models. However, it is crucial to not just provide a cellular model with a disease-specific phenotype, but to also deliver the proof-of concept for a screening assay. Although the feasibility of iPSC and other patient-derived cellular models like fibroblasts for drug discovery has been demonstrated^10,11^, the reproducibility of an assay remains the biggest challenge and is highly dependent on the ability of the people performing the cultivation and, in case of iPSC and precursor cells, the differentiation^12,13^. The use of automated processes can help to decrease assay variability. However, the translation of manual processes into an automated protocol still poses major challenges. Complex hand motions, especially those requiring a high degree of eye-hand coordination, are difficult to perform by a robotic system. Furthermore, skilled laboratory staff constantly evaluates each step visually and applies spontaneous changes to the protocol (for example changes of splitting ratio or adapting trypsinization time). Such spontaneous decision-making is not compatible with automated platforms and therefore, standardized protocols have to be developed. The degree of difficulty to adapt a protocol for automated use increases with the complexity of the culture system. While classical cell lines like HeLa cells require very little care and are easy to culture on a platform, cultivation of patient-derived primary cells and iPSC are more delicate. Moreover, the needs of different cellular models can differ substantially and each cellular model needs tailored protocols of cultivation. Several automated platforms that have been reported in recent years^14–20^ are usually highly specialized to perform one task or culture one cell type. However, academic research institutes and facilities typically deal with many research groups that use different cellular disease models and require a variety of assays depending on the disease phenotype foreseen for a screening. Considering the high investment for an automated platform for cell culture maintenance and screening, a flexible system is needed that can be used to culture different cell types, to run independent screening campaigns, and to perform a high number of different assays using different readouts.

Here, we present a comprehensive integrated automated platform designed to (i) maintain and expand different cell types (adherent and suspension cells), (ii) differentiate iPSC-based disease-models in 2D and 3D, and (iii) perform high throughput and high content screenings (HTS/HCS). We report protocols for automated cultivation and expansion of fibroblasts (as prototype for adherent cells growing in serum-containing medium), iPSC, small molecule-derived neural progenitor cells (smNPC) and differentiation of those cells into 2D dopaminergic neuronal culture and 3D midbrain-specific organoids. These protocols allow to produce sufficient cells for HTS/HCS purposes and can be adapted to meet special requirements of other cell types enabling novel precision medicine approaches across diseases.

## Results

### System setup

**Figure 1:**
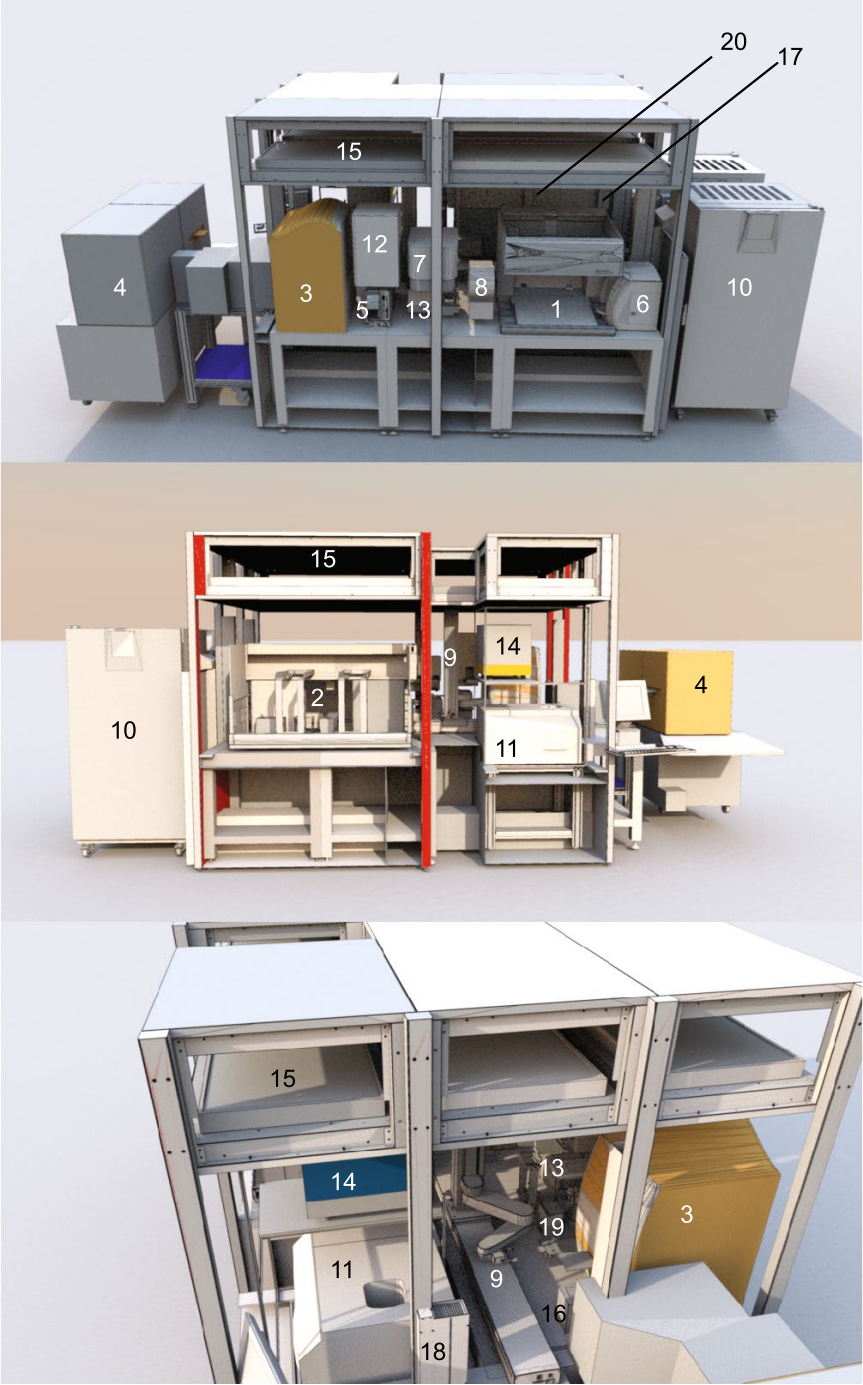
Schematic of the automated platform. (**1**) Liquid handler Biomek NX Span-8 including the plate shaker Shaking Peltier ALP and a tilting station Amplius (AIG) 3D Tilting ALP, (**2**) Liquid handler Biomek FXP (96- and 384-pipetting head including active wash-station and a BioShake 3000, (**3**) Acoustic droplet ejector Labcyte Echo550, (**4**) High content imager Yokogawa CV8000, (**5**) plate sealer Wasp, (**6**) Cell counter Vi-Cell XR12, (**7**) plate seal remover Nexus Xpeel, (**8**) Barcode printer and reader Scinomix Sci-Print MP2, (**9**) Precise SCARA robotic arm on rail, (**10**) 4 °C and 37 °C incubators Cytomat 24-C, (**11**) Sigma refrigerated centrifuge 6-16KR, (**12**) Tube de-capper Cap-it All, (**13**) Plate washer Biotek 405 LSHTV, (**14**) Multimode plate reader SpectraMax i3 Multi-Mode Platform and Minimax imager, (**15**) Custom made biosafety class 2 enclosure, (**16**) 2D-barcode reader Visionmate, (**17**) 4 deliiding modules, (**18**) 5 Tipbox labware feeders, (**19**) Regrip station, (**20**) 4 shelf labware hotel.

The system was designed to be flexible enough (i) to perform cell culture protocols of various cell types, (ii) to develop assays related to phenotypic readouts, and to translate these assays into high throughput / high content screenings (HT/CS) (Fig. 1). The platform is equipped with two pipette tip-based liquid handler with a pipetting range from 384 times 1 µl to eight times 1 ml to allow cell culture maintenance and expansion in 24-, 12-, 8-, 6-, and 1-well format, as well as handling of assay plates (384- and 96-well format). An automated incubator and fridge offer 451 and 358 positions, respectively, for cell culture plates, medium reservoir, and tube racks. An automated centrifuge was integrated to allow splitting of cells that grow in serum free conditions (no inactivation of enzymes like trypsin) or in suspension. Confluency assessment of cultured plates is performed on an integrated multi-mode microplate reader (SpectraMax i3 Multi-Mode Platform) that can also be used for colorimetric and luminescence readouts. Further instruments are an automated confocal high content imager (Yokogawa Cell Voyager 8000), an acoustic droplet ejection liquid handler (Echo550) for pipetting in nanoliter range, and several additional devices for plate handling, labware supply, and data and compound management. In total, the platform comprises of 20 different stations (Fig. 1, methods) that are all connected via a robotic arm on a railway.

### Automated assessment of confluency and cell distribution

**Figure 2:**
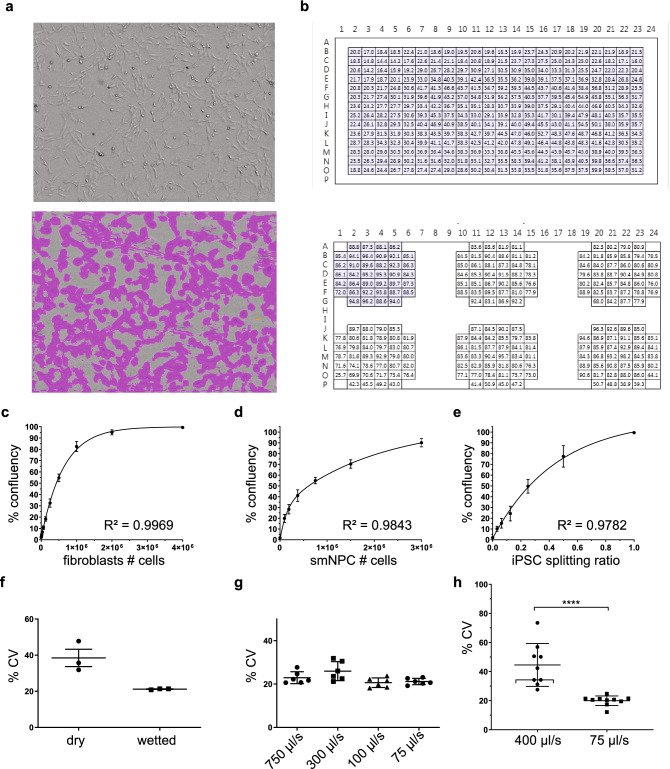
Quality control of cell seeding. (**a**) Fibroblast images taken with the multimode plate reader to determine confluency without (top) and with (bottom) mask highlighting areas covered by cells. (**b**) Acquisition mask for confluency measurement of a 1-well plate (top) and a 6-well plate (bottom). Each field represents a field of acquisition and numbers in the fields indicate percentage of confluency. (**c**–**e**) Non-linear curve fitting on confluency reads of serially diluted or split fibroblasts (n = 3) (**c**), smNPC (n = 7) (**d**), and iPSC (n = 4) (**e**). (**f**) Measurement of CV after dispensing test with fibroblast into dry or pre-wetted 6-wells. n = 3. (**g**) Measurement of CV after testing dispensing speeds with fibroblast in 6-wells. n = 6 (**h**) Measurement of CV after testing dispensing speeds with iPSC in 6-wells. n = 9, statistical test used: Mann–Whitney test.

Maintaining cells in culture over several passages goes beyond simple liquid handling to replace medium. After splitting the cells, there are two crucial parameters for maintaining quality of cell culture: confluency of cells in the destination wells and the distribution in the destination wells. To assess these two parameters we adapted the StainFree Cell Detection Algorithm of the SpectraMax i3 Multi-Mode Platform to the cell types and plate formats used in this study (Fig. 2a). All cell types were cultured and passaged in standard 6-well plates for maintenance and transferred to 1-well plates for expansion. To asses confluency and distribution in these plates we acquired 38 fields per well of a 6-well plate and 384 fields per 1-well plate and determined the percentage of area covered by cells (Fig. 2b). The average of all fields per well represents the confluency in the well and the coefficient of variance (CV) of these measurements was used as indicator of the cell distribution in the well. We validated the robustness of the image analysis by serial dilution experiments. Different numbers of fibroblasts (Fig. 2c) and smNPC (Fig. 2d) were seeded in 6-well plates and confluency was determined the next day. For iPSC, cell number cannot be used as indicator as they are passaged as clumps. Therefore, we have split 100% confluent wells in different ratios (Fig. 2e). The confluency reads reflected the increasing amounts of seeded cells/ cell clumps and non-linear curve fitting indicated high consistency of the analysis with R^2^ > 0.96 for all three cell types (Fig. 2b–d).

### Dispensing speed influences seeding homogeneity

In contrasts to the other cell types in this study, fibroblasts do not require coated plates to attach to the labware. However, when seeded in dry plates, the distribution of the attached cells was significantly less homogeneous compared to seeding into wells that were pre-wetted with water (Fig. 2f). Next, we assessed the optimal dispensing speed. Although, the different speeds had only minor effect on cell distribution, we decided to set 75 µl/s as default dispensing speed for all experiments, as it showed the most robust result (Fig. 2g). For iPSC 75 µl/s dispensing speed was tested against 400 µl/s and resulted in significantly more consistent colony distribution in the destination plates (Fig. 2h). Therefore, 75 µl/s was set as default speed and subsequently also used for smNPC dispensing.

### Fibroblast maintenance

**Figure 3:**
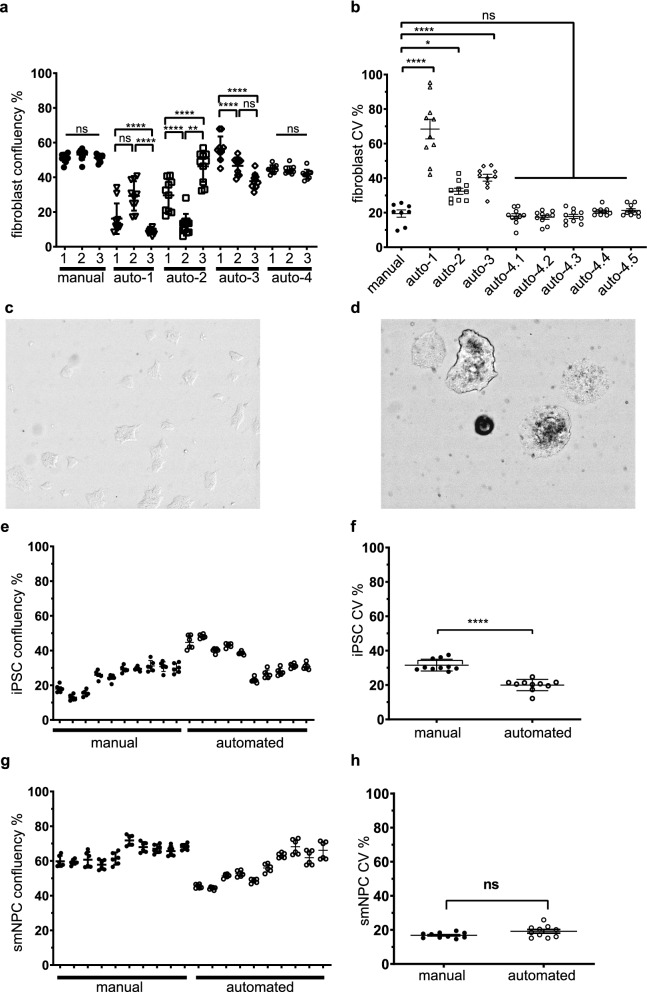
Automated cell cuture maintenance. (**a**) Confluency of fibroblasts 24 h post seeding in 6-well plates split either manually or with automated methods 1, 2, 3, or 4 into three destination wells. n = 10 statistical test used: one-way ANOVA with addhoc Tukey’s test. (**b**) CV of fibroblasts 24 h post seeding in 6-well plates split either manually or with automated methods 1, 2, 3, or 4 into three destination wells. After passaging with method 4 plates were either left unshaken (4.1) or shaken for 1 min (4.2) or 5 min (4.3) with a linear shaker, 3 min with an orbital shaker (4.4), or pivoted with the tilting station for 3 min (4.5). n = 10 statistical test used: one-way ANOVA with addhoc Sidak’s test. (**c**, **d**) Representative image of iPSC 24 h post splitting with EDTA (**c**), or dispase (**d**). (**e**) Confluency of 20 6-well plates of iPSC 24 h after splitting them either manually or with the automated method 1:7. (**f**) CV of iPSC from (e) n = 10 statistical test used: Mann–Whitney test. (**g**) Confluency of 20 6-well plates of smNPC 24 h after splitting them either manually or with the automated method 1:7. (**h**) CV of smNPC from (f) n = 10 statistical test used: Mann–Whitney test. *p < 0.05, **p < 0.01, ***p < 0.001, ****p < 0.0001.

When fibroblasts were passaged manually, they were treated with 500 µl trypsin in the well of a 6-well plate for 5 min. Subsequently, 1 ml medium was added and the cells are triturated with the pipette before a fraction of the cells (in this case a third for a splitting ratio of 1:3) was transferred to each destination well that have been prefilled with fresh medium. Finally, the destination plate was shaken in a shape of a cross before incubation (Supplementary Fig. 1a). When the platform was programmed to reproduce this protocol, the three destination wells originating from one source well displayed significantly different confluencies and were less confluent as manually split wells (Fig. 3a automated method 1, Supplementary Fig. 1a). The CV of the confluency was significantly higher compared to manually split wells (Fig. 3b automated method 1). We hypothesized that the fibroblasts sediment during the transfer step. To maximize the yield of collected cells after incubation with trypsin, the plate was tilted along the short edge and cells were subsequently aspirated from the lower edge of the well. We hypothesized that the transfer from this tilted plate directly to the destination wells led to uneven separation of cells and less homogeneous distribution in each destination well. To counteract this effect, we increased mixing steps with the pipette in the source well prior every aspiration step and collected the cells of each aspiration step into one well of a deep well plate. The cell suspension was mixed again before distributed among the destination wells (Supplementary Fig. 1a). Although this intervention improved the CV within each well (Fig. 3b automated method 2), it failed to improve the inter-well variability in terms of confluency (Fig. 3a automated method 2). To achieve homogenous confluency in all three destination wells, the trypsinated cells from one well were collected in a single well of a deepwell plate. Additionally the source well was flushed once more by dispensing fresh medium at the top edge of the tilted well and transferred to the collected cells. After several mixing steps the cell suspension was evenly divided between three new wells of the deepwell plate. The content of each well was then mixed again and seeded into prefilled destination wells (Supplementary Fig. 1a). This protocol increased the confluency in the destination wells, but the comparison of the three still showed significant differences (Fig. 3a automated method 3). The CV was also still significantly higher compared to manually split cells (Fig. 3b automated method 3). To improve the intra-well distribution, we decided to prepare the final volume of cell suspension we need for the destination well (1.5 ml) in the deepwell plate and seed the cells after additional mixing steps. Additional mixing steps were also added prior dividing the content of the first well of the deep well plate onto three wells (Supplementary Fig. 1a). With this final protocol, we achieved results that are comparable to manual splitting in terms of inter- and intra-well variability (Fig. 3a,b automated method 4, Supplementary Table 1). Next, we assessed whether we can improve the distribution of cells within a well by different shaking methods. We tested three different devices; a linear shaker, an orbital shaker, and pivoting the plate with the tilter. None of the shaking methods improved the distribution of cells further (Fig. 3b automated method 4–1 to 4–5). Hence, none of these devices is needed for fibroblast maintenance. However, one orbital shaker is needed on the platform, as detachment of trypsinized cells as well as distribution of coating solutions requires shaking.

### iPSC and smNPC maintenance

iPSC and smNPC require matrix coated plates to grow adherently. Plates were coated by the platform and were kept in the fridge of the system until being used. To split iPSC, we compared first the use of EDTA solution with the use of a neutral protease solution (Dispase) to split iPSC as clumps. To detach the colonies, iPSC were washed three times with PBS and then incubated with either detachment solution and, subsequently, the plates were shaken for 1 min before being tilted and cells being collected as described for fibroblasts. While EDTA is neutralized by addition of medium after the detachment and only one wash step is needed, dispase needs to be removed by at least two wash steps. Furthermore, treatment with EDTA and subsequent shaking of the plate to detach the iPSC disrupted the colonies more efficiently than treatment with dispase and resulted in smaller clumps (Fig. 3c). Hence, ten mixing pipetting steps were additionally needed to break the colonies when using dispase. Still, when split with dispase, the re-plated colonies were bigger than colonies split with EDTA and partially differentiated spontaneously. We concluded that traces of dispase might damage the replated iPSC and therefore increased the washing step to three cycles (Fig. 3d). However, even after three washes the high number of colonies with spontaneous differentiation remained. Adding more wash cycles would unreasonably extend the duration of the method. Therefore, we decided to use EDTA solution in the following protocols. As for fibroblasts, we used deepwell plates to collect the cell clumps, but the iPSC were additionally washed to remove residual detachment solution. After centrifugation the iPSC were resuspended and evenly distributed in seven with medium prefilled wells of the deepwell plate. In contrast to the fibroblast methods, we included only one mixing step during collection of the iPSC to avoid further dissociation of the clumps to single cell stage. Five independent iPSC lines were used to evaluate the maintenance splitting method. One 6-well was split to seven 6-wells to produce one entire plate for expansion and one more well for further maintenance (Supplementary Fig. 1b, Supplementary Table 2). The confluency in wells produced by the platform was significantly higher than in manually prepared plates and the destination wells generated from the same source well had similar levels of confluency (Fig. 3e). The passaging of iPSC on the platform significantly improved the distribution of colonies in the destination plates compared to manually split plates (Fig. 3f). Two iPSC lines that were cultured in parallel either manually or on the platform for 10 passages remained karyotypically stable and pluripotent, as determined by morphology, expression of pluripotency markers, and three germ layer differentiation (Supplementary Fig. 2a–d).

smNPC^21^ are a suitable cell type for automated processes as they (i) can be passaged in single cell state like fibroblasts, (ii) are fast proliferating, and (iii) differentiate efficiently into different types of neurons. Apart from the splitting ratio and a centrifugation steps to remove residual detachment solution, smNPC could be handled with the same techniques as fibroblasts (Supplementary Fig. 1c, Supplementary Table 3). Due to the high proliferation rate and the robustness, we adjusted the splitting ratio from 1:3 to 1:7. As for the other cell types, the automated method produced in each experiment (in total 3 independent lines were used) destination plates with consistently similar confluencies (Fig. 3g). The average confluency in the destination plates when split 1:7 was slightly lower with the automated protocol than in the manually prepared destination plates. There was no difference in CV between the two methods (Fig. 3h).

### Expansion of cells

**Figure 4:**
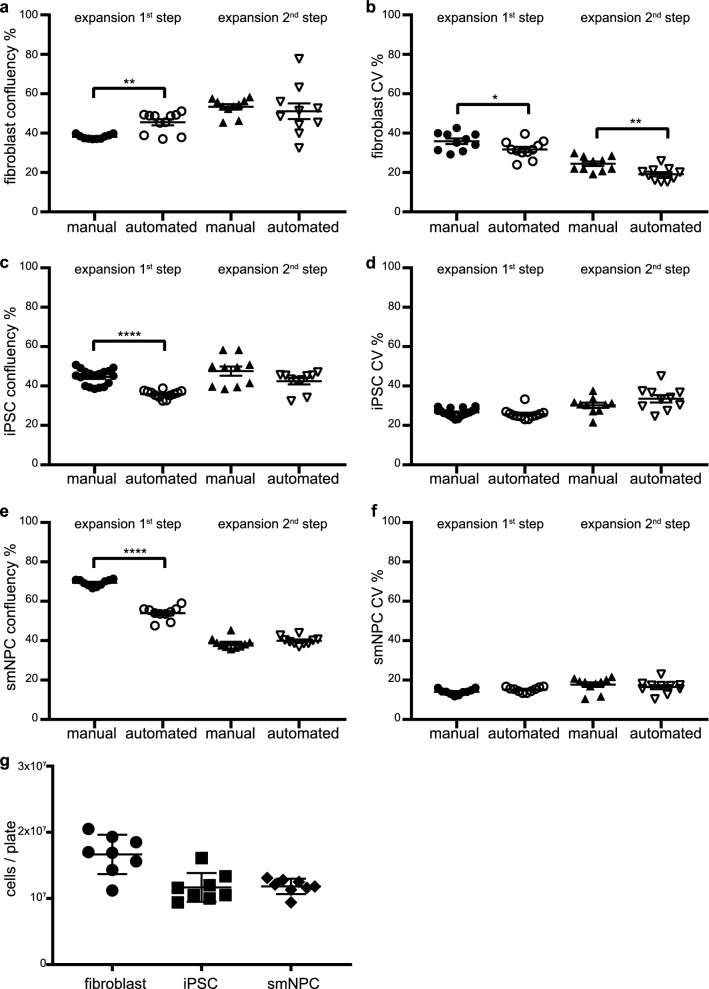
Automated cell culture expansion. (**a**) Confluency of fibroblasts 24 h post splitting for 1st and 2nd expansion step. n = 10, 1st step = Mann–Whitney test; 2nd step = two-tailed t-test. (**b**) CV of fibroblast plates from (**a**). two-tailed t-test. (**c**) Confluency of iPSC 24 h post splitting for 1st (n = 12) and 2nd (n = 10) expansion step. 1st step = two-tailed t-test; 2nd step = Mann–Whitney test. (**d**) CV of iPSC plates from (**c**). 1st step = Mann–Whitney test, 2nd step = two-tailed t-test. (**e**) Confluency of smNPC 24 h post splitting for 1st and 2nd expansion step . n = 10, two-tailed t-test. (**f**) CV of smNPC plates from (**e**). n = 10, 1st step = Mann–Whitney test; 2nd step = two-tailed t-test. (**g**) Number of cells counted from confluent 1-well plates. n = 8. *p < 0.05, **p < 0.01, ****p < 0.0001.

In the first step of moving forward from maintaining the cells to prepare enough material for screening, fibroblasts were expanded by stepwise splitting them to plates with bigger growth area. In the first step, three confluent wells of a 6-well plate were passaged together into one 1-well plate, which corresponds approximately to a splitting ratio of 1:3. Detached cells from each well were first collected in one well of a deepwell plate, respectively, diluted with fresh medium and underwent several pipette mixing steps. The cell suspension was then evenly distributed among eight prefilled wells of the deepwell plate. Subsequently, the cells were seeded to a 1-well plate using all eight 1 ml-channels of the liquid handler (Supplementary Fig. 1d, Supplementary Table 4). To distribute the cell suspension evenly the plates were shaken for 1 min at 1000 rpm. Plates generated by the platform were slightly more confluent (Fig. 4a) and showed a slightly better cell distribution compared to manually produced plates (Fig. 4b). In the second step of expansion the 1-well plates from the passage before were split into three new 1-well plates. Detached cells were collected in a 300 ml reservoir and diluted with medium to reach the final seeding volume (Supplementary Fig. 1e, Supplementary Table 5). Prior seeding from this reservoir the cell suspension was mixed by pipetting using the 96-channel pipetting head, which was also used for seeding. The automated method produced plates that were in terms of confluency comparable to manually prepared plates (Fig. 4a) and had a slightly yet significantly lower CV (Fig. 4b).

To expand iPSC, two confluent wells of a 6-well plate were passaged into a 1-well plate using EDTA solution, which corresponds approximately to a splitting ratio of 1:5. The procedure is similar to the fibroblast splitting with the exception that mixing steps before aspiration of cells were kept to a minimum to avoid dissociation into single cells (Supplementary Table 6). The results of this method were comparable with manually split plates (Fig. 4c,d). For further expansion, confluent 1-well plates were split into five new plates. To avoid dissociation of iPSC into single cells the detached iPSC clumps were not collected into a reservoir after washing and mixed with the 96-channel head, but were directly collected and distributed among eight wells of a deepwell plate. After washing to remove residual EDTA solution resuspended iPSC were transferred to five prefilled destination plates (Supplementary Fig. 1f). Again, mixing steps were kept to a minimum of four steps (Supplementary Table 7). The average confluency and the distribution of colonies in the destination plates were similar to those split manually (Fig. 4c,d). For the expansion of smNPC the same protocols as for fibroblasts were used, with the difference that cells were always washed in deepwell plates after detachment to remove residual detachment solution. As smNPC have a faster cell cycle and tolerate seeding in low cell concentration the splitting ratio was higher than for fibroblasts. In the first round of expansion, each confluent 6-well was split into one 1-well plate, which corresponds approximately to a ratio of 1:9 (Supplementary Table 8). Destination wells generated by the automation had lower confluency (Fig. 4e) and similar CV (Fig. 4f) when compared to manually split wells. To further expand the cells, confluent 1-well plates were split into six destination plates as described for fibroblasts (Supplementary Table 9). The confluency and cell distribution in these plates was comparable with manually split plates (Fig. 4e,f).

A confluent 1-well plate of fibroblasts (mean = 94.5%), iPSC (mean = 86.2%), and smNPC (mean = 89.3%) contained in average over 1.67 × 10^7^, 1.18 × 10^7^, and 1.17 × 10^7^ cells, respectively (Fig. 4g). Thus, ten plates produced by the automated method are sufficient to expand fibroblasts to prepare 19, and smNPC and iPSC to prepare 15 384-well plates containing 20,000 cells/well for HTS/HCS purposes.

### Automated neuronal differentiation

**Figure 5:**
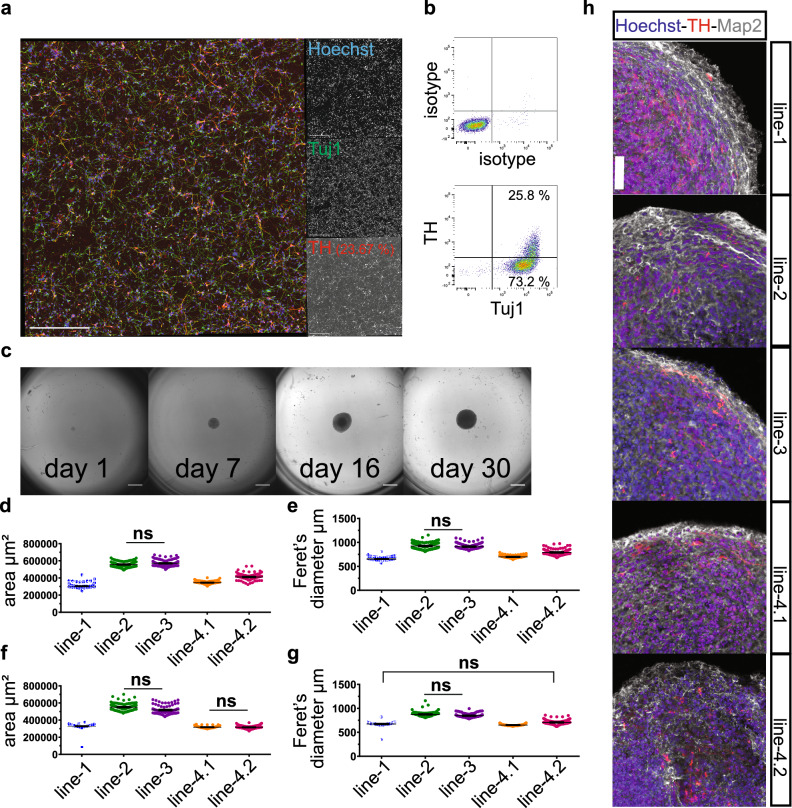
Automated neural differentiation in 2D and 3D. (**a**) Representative image of neurons produced with an automated 2D differentiation protocol for midbrain dopaminergic neurons. Cells were stained with HOECHST (blue, nuclei), anti-Tuj1 antibodies (green, pan-neuronal marker), and anti-tyrosine hydroxylase (TH) antibody (red, dopaminergic neurons). Scale bar = 200 µm. (**b**) Exemplary flowcytometric analysis of Tuj1 and TH expression in neurons from the same differentiation as in (**a**). (**c**) Bright field images of organoids from the same differentiation at indicated days of generation. Scale bar = 500 µm. (**d**–**g**) Analysis of organoid sizes extracted from brightfield microscopy images. Experiments between which the measured parameter was not significantly different are marked (ns). The observed differences between all other experiments were statistically significant (p < 0.05). (**d**) Organoid area at the end of differentiation (day 30). Five independent differentiations each with 96 organoids are shown. Differentiations were performed at different times. Kruskal–Wallis test followed by Dunn’s multiple comparison test. (**e**) Feret’s diameter of the organoids from (**d**). Kruskal–Wallis test followed by Dunn´s multiple comparison test. (**f**) Organoid area at the end of differentiation from a replication experiment. Differentiations were performed simultaneously. Kruskal–Wallis test followed by Dunn’s multiple comparison test. (**g**) Feret’s diameter of the organoids from (f). Kruskal–Wallis test followed by Dunn’s multiple comparison test. (**h**) Representative immunofluorescence images (maximum intensity projection) of organoids from (**d**) stained with HOECHST (blue, nuclei), anti-Map2 antibodies (white, pan-neuronal marker), and anti-tyrosine hydroxylase (TH) antibody (red, dopaminergic neurons). Scale bar = 50 µm.

The outcome of a cellular phenotypic screenings can be highly dependent on the cell type used. When using a disease specific cellular phenotype as readout, it might be necessary to perform the screening on the right target cells. smNPC can be differentiated into different neural lineages. As a prototype for automated in vitro differentiation of smNPC, we have established the automated protocol to generate midbrain-specific dopaminergic neurons^21^. We took advantage of the established protocols for smNPC expansion, as the proliferation rate during this differentiation increases dramatically in the first ten days before the cells become post-mitotic. The differentiation starts with a confluent well of a 6-well plate. The cells are passaged into a matrix coated 1-well plate and subjected to differentiation medium. The cells grow confluent within three to four days and are subsequently split. A second split is performed around day eight of differentiation to expand the cells to a total of nine 1-well plates. At day ten, the medium is changed to maturation medium and the neurons mature in these plates for a minimum of 11 days up to 30 days (Fig. 5a). The goal is to maintain these cells at high confluency until they are prepared for a screening in assay plates. If longer maturation of the neurons is required, the cells have to be passaged again every 21 to 30 days because degradation of the coating matrix reduces the adherence of the cell layer. Seven days prior an assay, neurons are detached from the 1-well plates, collected, and diluted in one 300 ml reservoir to generate a final seeding solution for assay plates (Supplementary Fig. 1g). Two independent differentiations until day 21 were performed with this method. The cells were obtained from the experiments to establish smNPC maintenance protocols and had been cultured on the platform for at least 5 passages before differentiation. The differentiations resulted in cultures comprised of > 95% neurons (Tuj1 positive) of which ~ 25% were dopaminergic neurons (TUJ1/TH positive) as determined by immunocytochemistry in the prepared assay plates (Fig. 5a) and in parallel by flow cytometry (Fig. 5b).

### Automated generation of midbrain specific organoids

Differentiated 2D cultures of complex tissues like the brain have limitations to capture disease phenotypes, as they do not recapitulate the physiological 3D architecture and cell–cell-connectivity of the organ. 3D organoids have become the state-of-the-art model to overcome these limitations. We have adapted a protocol for midbrain-specific organoid differentiation for automation as proof of concept for the generation of screenable cultures^22^. Organoids are usually either embedded in an extracellular matrix that serves as scaffold or cultured in low adherence plates, in which the cells self-organize. Only the latter is suitable for automated platforms, as embedding requires constant manual intervention to ensure that organoids are remaining in the center of the matrix droplet. We used 3000 smNPC per well in an ultra-low adherence 96-well plate to generate organoids. The cells were kept for eight days in smNPC medium to allow spheroid formation. At day eight, the medium was changed to differentiation medium. At day 30, the size of organoids ranged from 300,000 to 570,000 µm^2^ (Fig. 5c). It is crucial for screening purposes that organoids within one batch have similar size, as the effect of a compound could vary depending on the size of an organoid. Five different smNPC lines, two of which were different clones of the same donor (line-4.1 and line-4.2), were used for automated organoid generation in 96-well format. Two parameters of size were extracted from brightfield images (Fig. 5c) at day 30; (i) the area of the organoid and (ii) the Feret’s diameter. The size distribution of organoids of each line was very homogeneous with a CV between 5 and 9% for the area (Fig. 5d, Supplementary Fig. 3a) and between 4–8% for the Feret’s Diameter (Fig. 5e, Supplementary Fig. 3b) and thus suitable for compound screenings. However, the comparison between the lines showed significantly (p < 0.01) different sizes, except between lines 2 and 3. To determine whether these differences were due to experimental procedures (each differentiation was performed independently) or due to biological variation between the lines, we simultaneously generated another batch of each line. The organoids of this second generation also had a similar homogenous size distribution within each line with a CV of under 10% for both parameters (Fig. 5f,g, Supplementary Fig. 3a,b). The organoid size of each line was comparable to the organoids of the same line from the first differentiation. When both batches are analyzed together, the CV of each line remained for both parameters under 10%, with the exception of line 4.2, for which the CV of the area reached 15.56% (Supplementary Fig. 3a,b). The differences between the lines observed in the first batch of differentiations is recapitulated by the second batch (Fig. 5f,g), suggesting that the differences are indeed due to biological variation between the lines. Interestingly, the two clones from the same donor give rise to organoids of comparable size, arguing for specific effects related to the underlying genetic background. The successful differentiation into midbrain specific organoids was validated by staining for the neuronal marker Map2 and the dopaminergic marker TH (Fig. 5h).

## Discussion

Phenotypic screening is experiencing a renaissance, which is mainly driven by the availability of advanced patient-derived cellular models due to the recent advances of stem cell technology. With their unique access to patient material and their expertise in disease modeling, academic institutions are well positioned to take the lead in providing disease-specific cellular models for drug discovery. However, to attract public–private partnerships with industrial collaborators, it is not enough to identify a disease relevant phenotype, but to also provide a proof of concept by successful assay development and an initial screening to demonstrate that the identified disease-associated cellular phenotypes can be used for HTS/HCS purposes^10,11,23–26^. Reproducibility of assays in patient-derived cellular models remains the biggest challenge, especially when performed by different researchers or laboratories. Besides factors that are not related to the handling of cells like the different genetic background of donors, minor differences in cultivation and differentiation can have a big effect on primary cells and iPSC and lead to assay variability. In a five center study laboratory specific variability of molecular phenotypes and differentiation outcome was detectable although all five laboratories were provided with the two same iPSC lines and a detailed standard operating procedure^27^. Differences between the laboratories concerning number of passages during differentiation, feeding volume, feeding at weekends, and freezing of neural progenitors lead to variability that even masked genotypic effects that separated the two provided iPSC lines phenotypically. Variability is also reported to occur due to differences in medium composition and feeding cycles^28–30^. Even the culture conditions of iPSC can influence the outcome of a differentiation performed 4 weeks later^31^. Automated platforms can help tackling many of these challenges by introducing more consistency in experimental procedures. Therefore, more and more academic institutions are acquiring automated systems, but the vast majority of reported automations are single task platforms, capable of either culturing one cell type or performing one assay^16,17,19,20,32–36^. In contrast to those platforms, our custom-made platform is designed to handle various cell types and perform different assays. The capability to implement different patient-based cellular models is of increasing importance in the light of expanding translational research in academia and increasing numbers of inter-institutional screening facilities^37,38^. The platform is built on existing state-of-the-art devices combining classical tip-based pipetting (mainly used for cell handling) and contactless acoustic droplet ejection. The primary readout device is a high-end laser-based automated four-camera confocal spinning-disc microscope with dispensing capability allowing for kinetics readouts. Having all devices integrated via a robotic arm allows the implementation of complex protocols tailor fit for any cell type. An automated centrifuge is essential for protocols of cells growing in suspension or serum free. The multimode plate reader can be used for confluency measurements and for readouts with any luminescence, fluorometric or colorimetric assay. The acoustic droplet ejection system is used for cost-effective tipless compound dispensing, but can also be used to prepare HTS-qPCR^39,40^. However, manual protocols for cell culture need to be adapted for automation. Simple copying of manual steps by the liquid handler might not provide satisfying results as shown here for fibroblast splitting (Fig. 3a,b). Special protocols are required to ensure satisfactory and robust results. Here, we present protocols for (i) fibroblasts and smNPC as prototypes for adherent cells growing with or without serum, (ii) for iPSC as prototype for stem cells, and (iii) midbrain-specific organoids as prototypes for suspension cultures and 3D cultures. Cost effective protocols can be established even for delicate cells like iPSC. While the use of Rho-associated protein kinase (ROCK) inhibitor to ensure survival of the cells after passaging is expensive, it is still a common feature on many platforms^33,34,36^. Here, we developed a protocol free of ROCK inhibitor use (Fig. 3c,e,f). All tested cell types were consistently and efficiently expanded and differentiated to provide enough material for HTS/HCS campaigns with most commercial compound libraries. The type of automated platform described here provides a service portfolio that addresses the needs of translational medicine of diverse groups of research laboratories as they are usually found at academic institutions by integration and thereby providing important interfaces for public–private partnerships for successful drug development.

## Methods

### Platform description

The automated platform is a fully integrated system comprised of 20 stations (Fig. 1) and is controlled by SAMI EX Win7 automation controller software from BeckmanCoulter. All stations are connected via the SCARA Robot Extended Z, Extended Reach that is mounted on the SCARA robot 2.0 rail. Medium reservoirs and coated plates are stored at 4 °C in a Cytomat 24 C450 (ThermoFisher) incubator that is equipped with 10 Cytomat Stacker 50 mm (with 10 positions for max. labware height of 45 mm like deepwell plates), 6 stacker 33 mm (with 15 positions for max. labware height of 28 mm like 6-well plates), and 8 stacker 23 mm (with 21 positions for max. labware height of 18 mm like 96-well plates). Cells are incubated at 37 °C in a Cytomat 24 C1050 (ThermoFisher) incubator that contains 1 Cytomat Stacker 50 mm (with 10 positions for deepwell plates or reservoirs), 7 stacker 33 mm (with 15 positions for max. labware height of 28 mm like 6-well plates), and 16 stacker 23 mm (with 21 positions for max. labware height of 18 mm like 96-well plates). Liquid handling for cell culture maintenance is mainly performed on the Biomek NXp Span-8 Laboratory Automation Workstation (BeckmanCoulter). Each of the eight channels can pipette up to 1 ml and two positions on deck are occupied by the plate shaker Shaking Peltier and the plate tilting device Amplius (AIG) 3D Tilting ALP for BFX/BNX. The cell counting device Vi-CELL XR 12 (BeckmanCoulter) is attached to the Biomek NXp Span-8. The second tip-based liquid handler is a Biomek FXp Dual Arm System (BeckmanCoulter) with a Biomek 96-, and a 384- Channel Disposable Tip Pipetting Head that have a pipetting range from 1 to 200 µl and1 µl to 30 µl, respectively. The plate shaking device BioShake 3000 is occupying one position in this liquid handler. Both liquid handling robots are equipped with an active tip washstation that are used for liquid disposal. The acoustic droplet ejector Echo550 (Labcyte) completes the set of liquid handlers. It is mainly used for tipless, contactless transfer of compounds in nanoliter range for HTS purposes. Labware can either be delidded on deck by the Biomeks or on one of the 4 delidding modules ‘Single Pos. Lid Storage SAMI EX 4.1 SCARA Integration´. To provide the system with new labware, the labware can either be placed manually in the incubators or on a 4-shelf deck hotel. Pipetting tips are provided by three labware feeders that can each hold either up to 9 boxes of 1 ml tips or 19 boxes of 96- or 384 tips. Two additional labware feeder are used for retrieved tip boxes after completion of a protocol. All centrifugation steps are performed in the Sigma Refrigerated Centrifuge 6-16KR. Further devices for compound and plate management are the Wasp plate sealer (kbiosytems), the plate seal removal system Nexus Xpeel (Brooks life sciences), the barcode printer/reader Sci-Print MP2 (Scinomix), the tube (de-)capping device Capit-All (ThermoFisher), the 2D code reader VisionMate (ThermoFisher), the plate washer 405 LSHTV (Biotek), and a regrip position next to the Scara robot rail. The multi-mode plate reader SpectraMax i3 Multi-Mode Platform and Minimax imager (Molecular Devices) is used for confluency readings and the high content confocal imager CellVoyager CV8000 (Yokogawa) is used for screening campaigns with image based readouts. All devices except the incubators, the labware feeders and the CellVoyager CV8000 are placed under a custom-made biosafety class II enclosure (Noroir). All devices of the platform can also be operated as stand-alone units.

### Pipetting templates and techniques

The software of the two Biomeks allows to establish pipetting templates and pipetting techniques to control factors like aspiration and dispensing speed, movement of tips during pipetting, and mixing speed and frequency. These factors were optimized for the different liquids and cell types used in this study and are shared upon reasonable request.

### Consumables

Adherent cells were cultured in 6-well plates (Nunc 6-well multidish, 128 × 86 mm, Vwr) or 1-well plates (Omnitray 165218 1-well plate, VWR). 96-deepwell plates (Corning Storage plates and blocks, 96-well, V-bottom, Corning) or reservoirs (Reagent reservoirs, single-well, Axygen, VWR) were used to collect detached cells, wash step or to provide solutions. All transfers were performed with either Span-8 P1000 (Barrier, Presterile) tips, or BIOMEK AP96 P250 TIPS (STERILE BARRIER), or Biomek AP384 P30XL Tips (Sterile) (all BeckmanCoulter). Fibroblasts were cultured in DMEM supplemented with 1% Penicillin–Streptomycin 100 × and 10% FBS and detached for passaging by treatment with Trypsin–EDTA (0.25%), phenol red (all LifeTechnologies). iPSC were cultered in self-made E8 medium (DMEM/ Ham’s F12 + HEPES supplemented with 1% Penicillin–Streptomycin 100x, 1% ITS-supplement (all LifeTechnologies), 64 mg/l L-ascorbic acid-2-phosphate magnesium (Sigma-Aldrich), 10 µg/l FGF2 (R&D Systems), 2 µg/l TGF-β1 (Peprotech), 100 ng/ml Heparin and 10% mTeSR (Sigma-Aldrich)). To detach iPSC they were either incubated in PBS/ 0.5 mM EDTA or in iPSC medium containing 4 units/ml Dispase (Neutr. Protease) (Cell Systems). smNPC were cultured in N2/B27 medium with different supplements for maintenance and differentiation into midbrain dopaminergic neurons as previously described^21^.

### Plate coating

Matrigel Growth Factor Reduced (GFR) Basement Membrane Matrix (Corning) or Geltrex LDEV-Free, hESC-Qualified, Reduced Growth Factor Basement Membrane Matrix (Gibco) were diluted 1:100 in DMEM/F12 (Gibco) in a reservoir and loaded on the system via the 4 °C incubator. To coat 6-well plates, 1 ml coating solution was transferred with the Biomek NXp Span-8 into each well and the plate was shaken for 1 min at 1500 rpm on the Shaking Peltier to reach a 100% coverage of the well surface. To coat 384-well plates, 30 µl/well were transferred with the Biomek FXp and the plate was shaken for 2 min at 2700 rpm on the BioShake. Shaking at lower speed or centrifugation up to 5 min did not remove all air bubbles from the bottom of the wells and, hence, led to incomplete coating of the surface.

### Cell culture maintenance and expansion

Step by step descriptions of the successfully established final protocols for passaging the cells can be found in the supplemental information (Supplementary Tables 1–9). The duration of each protocol is depending on how many wells are processed in one run. Because most of the time is consumed by the transport of labware, the duration of a protocol does not increase linearly with increasing numbers of wells to be processed. The timing of the splitting protocols for one cell line and an optimized timing (combining maximum numbers of cell lines on the source plate) can be found in the supplemental information (Supplementary Table 10). Cell feeding was performed on the Biomek NXp Span-8. Medium reservoirs were transported from the 4 °C incubator to the 37 °C incubator 1 h prior feeding. Plates and medium reservoirs were retrieved from the incubator and transported to the Biomek NXp Span-8 by the SCARA robot, which also retrieved one tip box from the labware feeder. Plates were delidded on deck and the cell plate was placed on the tilting device. Medium was aspirated from the tilted plate and discarded into the wash station. Fresh medium was transferred from the reservoir to the cell plate. In all protocols, pipette tips were replaced after each step, except for repetitive steps like dividing the content of one well among different destination wells. Cells in 6-wells were fed with 1.5 ml and 1-well plates with 12 ml medium. Fibroblasts were fed every three days, iPSC daily, and smNPC and neurons every other day.

### iPSC characterization

Two iPSC lines were split 1:4 at the start of the experiment in two wells of two 6-well plates, respectively, and the rest of the cells was used as starting reference (passage 0) for karyotypic analysis. One of the six well plates was cultured manually for ten passages and the other on the platform. After 10 passages, automatically cultured cells were karyotyped again. The KaryoStat service (ThermoFisher SCIENTIFIC) was used for karyotypic analysis of iPSC. The ability of iPSC to differentiate into all three germ layers was tested after 10 passages by using the Human Pluripotent Stem Cell Functional Identification Kit (R&D Systems) according to manufacturer’s instructions. For immunocytochemical staining of pluripotency markers, iPSC (after ten passages of automated or manual culture) were seeded on cover slips 3 days prior fixation in 4% PFA for 15 min at room temperature. Fixed iPSC were stained using standard immunochemistry staining protocol (see analysis of neuronal culture). Primary antibodies were used at following dilutions: mouse anti-Oct3/4 (clone C-10, Santa Cruz) 1:200, goat anti-SOX2 (polyclonal Y-17, Santa Cruz) 1:200, rabbit anti-NANOG (polyclonal ab21624, Abcam) 1:250. All secondary antibodies were used at a dilution of 1:200.

### Microscopy and image analysis of neuronal culture

Neurons differentiated with the automated protocol were split at day 15 of differentiation into CellCarrier-384 Black, Optically Clear Bottom plates (Perkin Elmer) at a concentration of 25,000 cells/well. Cells were fixed with 4% PFA for 15 min at room temperature and washed 3 × with PBS. Cells were blocked with 0.4% Triton-X + 10% Goat Serum + 2% BSA in PBS for one hour. Primary antibodies (anti-Tubulin β 3 [clone TUJ1], BioLegend; anti-TH AB152 [polyclonal], Merck) were diluted 1:1000 in the same solution but with only 0.1% Triton X-100 and were incubated for 24 h at 4 °C. Secondary antibodies and Hoechst were applied for 1 h after washing with PBS and subsequently the cells were washed again and kept in PBS for imaging. Fluorescence images were acquired on an Opera QEHS spinning disc microscope (was previously part of the platform before being replaced by the Yokogawa CV8000) using a 20 × water immersion objective with numerical aperture 0.7 and three sequential exposures with binning 2. The first channel was excited with a 561 nm laser and detected behind a 600/40 bandpass filter. The second channel was excited with a 405 nm laser and detected behind a 450/50 bandpass filter. The third channel was excited with a 488 nm laser and detected behind a 520/35 bandpass filter.

Image analysis was performed using Matlab 2017b (Mathworks). Code snippets are given in Courier New Font. Nuclei were segmented using preprocessing with a difference of gaussians imfilter(ImNuc, fspecial('gaussian', 10, 2), 'symmetric')—imfilter(ImNuc, fspecial('gaussian', 60, 20), 'symmetric'), and thresholded (> 50). Nuclei splitting via shape, was implemented by euclidean distance transform: watershed(imhmin(imcomplement(bwdist(~ NucMask)), 1)). This splitting stencil was applied to the dilated nuclei mask imdilate(NucMask, strel('disk', 10)) in order to define single cell regions of interest (ROI) for image feature extraction. For the segmentation of neurons, TH respectively Tuj1 channels were preprocessed with a difference of gaussians highlighting fine structures imfilter(ImNeuron, fspecial('gaussian', 11, 1), 'symmetric')—imfilter(ImNeuron, fspecial('gaussian', 11, 3), 'symmetric') and a second difference of gaussians highlighting larger structures imfilter(ImNeuron, fspecial('gaussian', 99, 3), 'symmetric')—imfilter(ImNeuron, fspecial('gaussian', 99, 11), 'symmetric') and thresholded (THfine > 3, THbig > 10, Tuj1fine > 2, Tuj1Big > 3). For both channels, additional global thresholds were applied to the median filtered images (THmedian > 50, Tuj1median > 20). The final masks for TH respectively Tuj1 were defined via boolean OR operation between the above described masks: Mask = MaskLocalFine|MaskLocalBig|MaskGlobal. The neuronal mask was defined as NeuroMask = THMask|Tuj1Mask. Boolean AND operation of the above mentioned nucleus centered ROIs with the neuronal mask excluded ROIs outside of neurons. To identify TH positive cells, the TH background defined as the mean TH fluorescence intensity outside of nuclei was subtracted from the raw TH channel. For that image, the threshold for TH positive cells/ROIs was set to 20.

### Flow cytometry

Neuronal cultures were detached using Accutase and fixed in 4% PFA for 15 min at room temperature (RT). 5 × 10^5^ cells were washed three times with PBS/5% BSA and subsequently permeabilized for 20 min on ice in saponin buffer (0.05% saponin/1% BSA/PBS). Permeabilized cells were kept for 30 min in PBS/5% BSA at RT for blocking. After blocking, cells were pelleted and resuspended in 100 µl saponin buffer containing primary antibodies at a dilution of 1:100 (anti-TH AB152 [polyclonal], Merck) and 1:200 (anti-Tubulin β 3 [clone TUJ1], BioLegend) and incubated for 30 min at 4 °C. Subsequently, cells were washed three times with PBS/5% BSA and resuspended and stained for 30 min at 4 °C in 100 µl PBS containing secondary antibodies at a dilution of 1:200. After three more washes with PBS/ 5% BSA cells were resuspended in PBS and analyzed in the BD LSRFortessa flow cytometer. Measurements were analysed using FlowJo_V10 software.

### Organoid differentiation

smNPC suspension in smNPC medium with a concentration of 30,000 cells/ml was provided in reservoir and loaded onto the platform via the 37 °C incubator. Cell suspension, a 96-tip box and a Ultra-Low Attachment Multiple 96-well plate (Corning Costar) are transported to the Biomek FXp Dual Arm System. The cell suspension is mixed twice with the 96-pipetting head with a volume of 100 µl/tip before 100 µl suspension are transferred to each well of the ultra-low adherence plate. Subsequently, the plate is centrifuged for 3 min at 900 rpm before sending the plate to the incubator. 70 µl of the medium in each well is replaced every other day for 14 days. After the aspiration of 70 µl on day 14 the wells are filled with 120 µl fresh medium. From this moment, feeding happens every 4 days and 120 µl/well are replaced each time. From day 0 to day 7 cells are cultured in smNPC medium. On day 8 medium composition is changed to organoid differentiation medium for 6 days and from day 14 onwards the organoids are cultured in maturation medium as previously described^22^.

### Organoid immunofluorescence staining

Staining of organoid sections was performed as previously described^41^. Organoids were fixed with 4% PFA overnight at 4 °C and washed 3 × with PBS for 15 min. They were then embedded in 3–4% low-melting point agarose in PBS. The solid agarose block was sectioned with a vibratome (Leica VT1000s) into 80 µm sections. The sections were blocked on a shaker with 0.5% Triton X-100, 2% BSA, and 5% normal goat serum in PBS for 90 min at RT. Primary antibodies were diluted in the same solution but with only 0.1% Triton X-100 and were incubated for 48 h at 4 °C. The following first antibodies were used: anti-mouse MAP2 (Millipore, 1:200) and anti-rabbit TH (Santa Cruz 1:1000). After incubation with the primary antibodies, sections were washed 3 × with PBS and incubated with the secondary antibodies and Hoechst in 0.05% Tween-20 in PBS for 2 h at RT and washed with 0.05% Tween-20 in PBS and Milli-Q water before they were mounted in Fluoromount-G mounting medium (Southern Biotech). The organoids were imaged using a Zeiss confocal microscope (LSM 710).

## Supplementary Information

Supplementary Information.
