# Supplementary material for: Integrated, automated maintenance, expansion and differentiation of 2D and 3D patient-derived cellular models for high throughput drug screening
Source: Sci Rep. 2021 Jan 14;11:1439. doi: 10.1038/s41598-021-81129-3 (PMC7809482; doi:10.1038/s41598-021-81129-3)
Supplement: Supplementary file 1 — Supplementary Information. [file 41598_2021_81129_MOESM1_ESM.docx]

**Supplementary Material**

**Integrated, automated maintenance, expansion and differentiation of 2D and 3D patient-derived cellular models for high throughput drug screening**

Ibrahim Boussaad^1,2^, Gérald Cruciani^1,2^, Silvia Bolognin^3^, Paul Antony^1,2^, Claire M. Dording^2,7^, Yong-Jun Kwon^2,4^, Peter Heutink^5^, Eugenio Fava^6^, Jens C. Schwamborn^3^, Rejko Krüger^1,2,7,8,^*

1 Translational Neuroscience, Luxembourg Centre for Systems Biomedicine,

University of Luxembourg, Luxembourg

2 Disease Modeling and Screening Platform (DMSP), Luxembourg Centre of Systems

Biomedicine (Biomedicine), University of Luxembourg and Luxembourg Institute of Health

(LIH), 6 avenue du Swing, L-4367, Belvaux, Luxembourg

3 Developmental Biology, Luxembourg Centre for Systems Biomedicine, University of

Luxembourg, Luxembourg

4 Oncology Department, Luxembourg Institute of Health (LIH), Luxembourg

5 German Center for Neurodegenerative Diseases (DZNE)-Tübingen & Hertie Institute for

Clinical Brain Research, Otfried Müller Strasse 23, 72076 Tübingen, Germany

6 German Center for Neurodegenerative Diseases (DZNE) - Core Research Facilities and

Services - Venusberg-Campus 1, Gebäude 99 D-53127 Bonn

7 Transversal Translational Medicine, Luxembourg Institute of Health (LIH), Luxembourg

8 Parkinson Research Clinic, Centre Hospitalier de Luxembourg (CHL), Luxembourg

* Correspondence should be addressed to R.K. (Rejko.krueger@uni.lu)


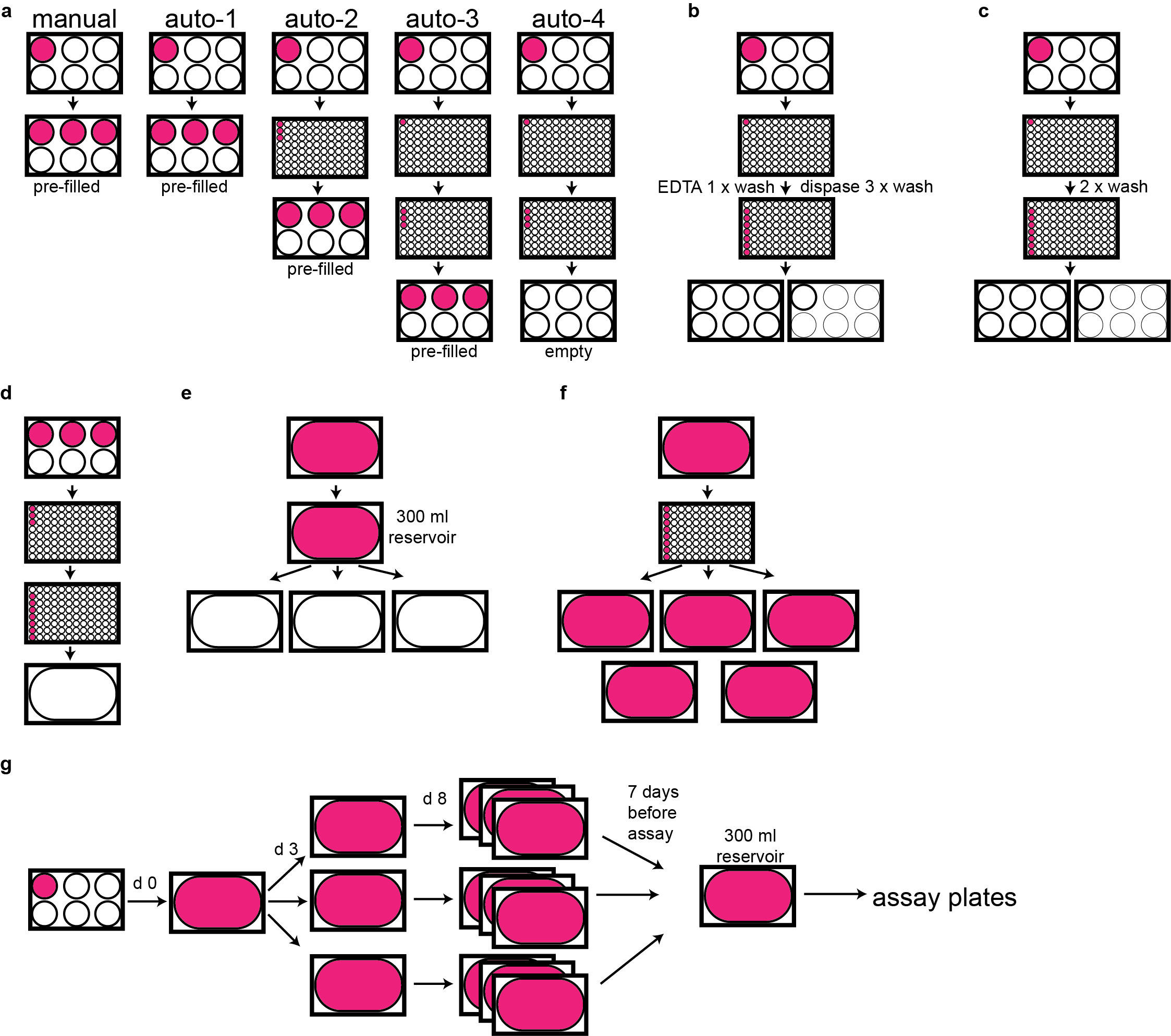


**Supplementary Figure 1: Schematic illustrations of automated processes.**

(**a-c**) Schematic illustrating automated methods of fibroblast (a), iPSC (b), and smNPC (c) splitting for maintenance purposes. Cells are passaged from a well of a 6-well plate to several wells partially via intermediate steps in a 96-deepwell plate. (**d,e**) Schematic of automated expansion protocols for fibroblasts from three 6-wells via a 96-deepwell plate to a 1-well plate and from a 1-well plate via a reservoir to 3 1-well plates. (**f**) Schematic illustration of iPSC expansion from one to five 1-well plates via a 96-deepwell plate (**g**) Schematic illustration of the 2D dopaminergic differentiation protocol of smNPC. The differentiation starts at day 0 in one 1-well plate and further passaging is performed at day 3, day 8 and 7 days prior the assay.


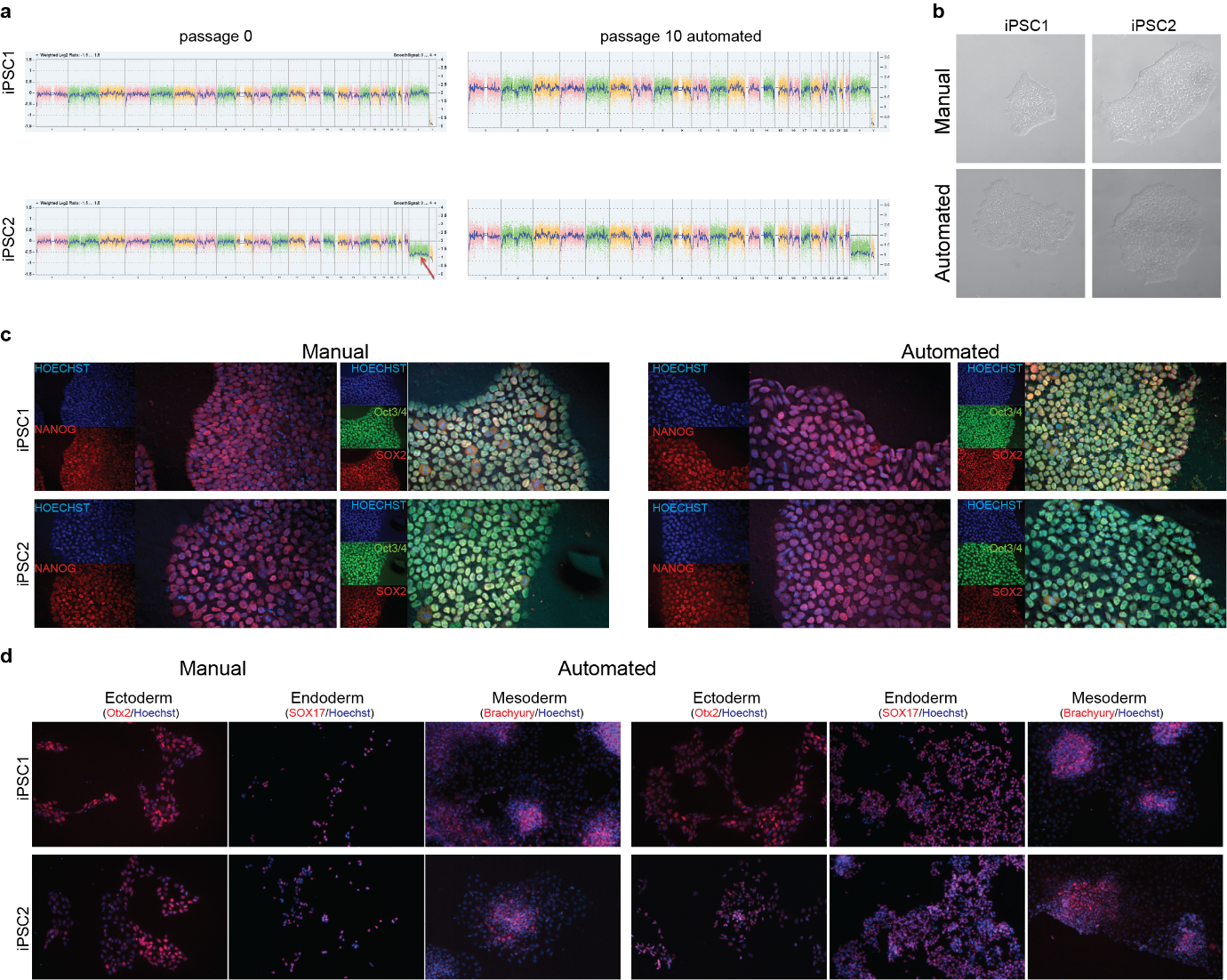


**Supplementary Figure 2: Characterization of automatically and manually cultured iPSC.**

Characterization of iPSC that were cultured for ten passages either manually or by the automated platform. (**a**) Whole genome view of the karyotypic analysis of iPSC showing the copy number state at the start (passage 0) and after ten passages of automated culture. Karyotypes remained stable. A mosaic chromosomal gain on the X chromosome (red arrow) that was detected in approx. 20 % of the cells of line iPSC2 at the start of the culture was not detectable anymore at passage 10. (**b**) Bright field images of iPSC colonies after ten passages of manual or automated culture. (**c**) Immunocytochemical staining of pluripotency markers NANOG, Oct3/4 and SOX2 after 10 passages of manual or automated culture. (**d**) Three germ layer differentiation of iPSC after 10 passages of manual or automated culture.


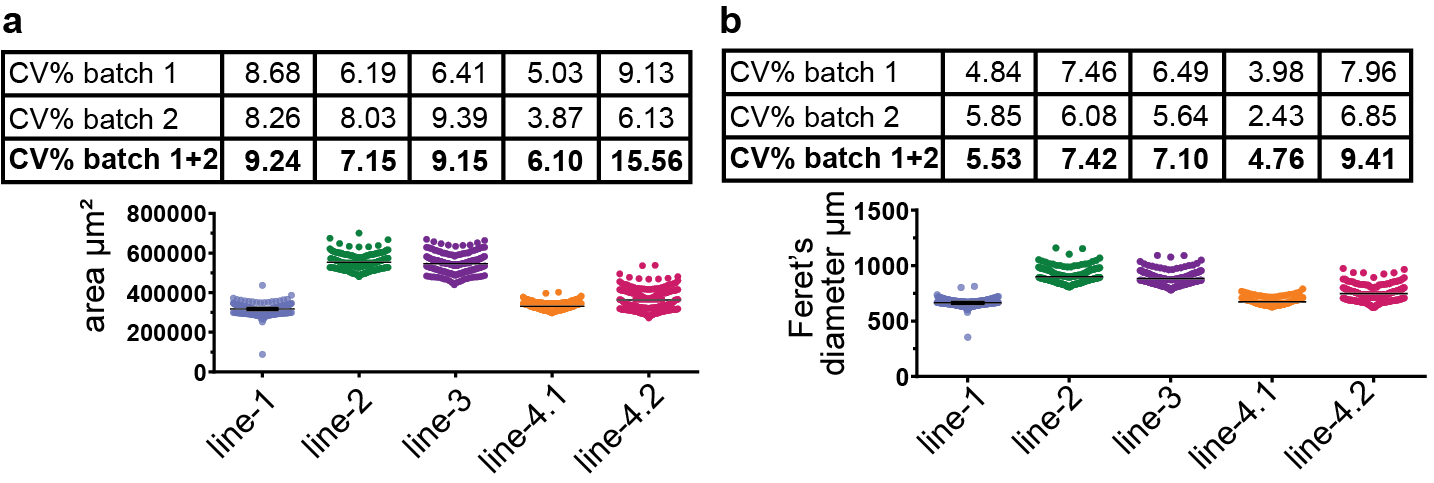


**Supplementary Figure 3: Inter-experimental and inter-cell line comparison of organoid size.**

(**a**) Area of organoids extracted from brightfield images. The first and second row of the table show the coefficient of variation of each experiment described in figure 5 d, f. The third row shows the coefficient of variation for each line when data from both batches are combined. All data points from both batches are plotted together in this graph. (**b**) Feret´s diameter of organoids extracted from brightfield images. The first and second row of the table show the coefficient of variation of each experiment described in figure 5 e, g. The third row shows the coefficient of variation for each line when data from both batches are combined. All data points from both batches are plotted together in this graph.

**Supplementary Table 1: Fibroblast passaging from one 6-well to three 6-wells**

| **Name** | **Labware** | **Position** | **Action** | **Plate tilted** | **Device** | **Volume in ml** |
| --- | --- | --- | --- | --- | --- | --- |
| Source plate | 6-well | NX Tilt | Emptying well to wash station | X | Span-8 pipette | 1.5 |
| Source plate | 6-well | NX Tilt | Add PBS |  | Span-8 pipette | 0.85 |
| Source plate | 6-well | NX Tilt | Emptying to wash station | X | Span-8 pipette | 0.85 |
| Source plate | 6-well | NX Tilt | Add Trypsin 0.25% |  | Span-8 pipette | 0.5 |
| Source plate | 6-well | Cytomat 37 °C | Incubation 5 min |  | Cytomat 37 °C |  |
| Source plate | 6-well | FX BioShake | Shaking 2000 rpm / 1 min |  | BioShake 3000 |  |
| Source plate | 6-well | NX Tilt | 8 Mixes and transfer to 96-DW to A1 | X | Span-8 pipette | 0.5 |
| Source plate | 6-well | NX Tilt | Flush with fresh medium | X | Span-8 pipette | 1.35 |
| Source plate | 6-well | NX Tilt | 8 Mixes and Transfer to 96-DW to A1 | X | Span-8 pipette | 1.35 |
| Source plate | 6-well | NX | 12 Mixes 800ul in 96-DW in A1 |  | Span-8 pipette | 0.8 |
| Collection plate | 96-DW | NX | Split in 3 wells to B1 to D1 |  | Span-8 pipette | 3x0.565 |
| Collection plate | 96-DW | NX | Add fresh medium to B1 to D1 |  | Span-8 pipette | 1.235 |
| Destination plates | 6-well | NX Tilt | Emptying water to wash station | X | Span-8 pipette | 1 |
| Collection plate | 96-DW | NX | 8 Mixes and Transfer B1 in 1^st^ Destination well |  | Span-8 pipette | 1.8 |
| Collection plate | 96-DW | NX | 8 Mixes and Transfer C1 and D1 to 2^nd^ and 3^rd^ destination wells |  | Span-8 pipette | 1.8 |
| Destination plates | 6-well | Cytomat 37 °C | Incubation |  | Cytomat 37 °C |  |

6-well = 6-well plate, 96-DW = 96-deepwell plate, NX = Biomek NXp,NX Tilt = Amplius (AIG) 3D Tilting device

**Supplementary Table 2: iPSC passaging from one 6-well to seven 6-wells**

| **Name** | **Labware** | **Position** | **Action** | **Plate tilted** | **Device** | **Volume in ml** |
| --- | --- | --- | --- | --- | --- | --- |
| Source plate | 6-well | NX Tilt | Emptying well to wash station | X | Span-8 pipette | 1.5 |
| Source plate | 6-well | NX Tilt | Add PBS to well |  | Span-8 pipette | 0.85 |
| Source plate | 6-well | NX Tilt | Empty PBS to wash station | X | Span-8 pipette | 0.85 |
| Source plate | 6-well | NX Tilt | Add PBS to well |  | Span-8 pipette | 0.85 |
| Source plate | 6-well | NX Tilt | Empty PBS to wash station | X | Span-8 pipette | 0.85 |
| Source plate | 6-well | NX Tilt | Add PBS to well |  | Span-8 pipette | 0.85 |
| Source plate | 6-well | NX Tilt | Empty PBS to wash station | X | Span-8 pipette | 0.85 |
| Source plate | 6-well | NX Tilt | Add EDTA to well |  | Span-8 pipette | 1 |
| Source plate | 6-well | Cytomat 37 °C | Incubate 6 min |  | Cytomat 37 °C |  |
| Source plate | 6-well | FX BioShake | Shake 2000 rpm / 1 min |  | BioShake 3000 |  |
| Source plate | 6-well | NX Tilt | Flush cells | X | Span-8 pipette |  |
| Source plate | 6-well | NX Tilt | 1 mix and Transfer from well to 96-DW (well A1) | X | Span-8 pipette | 0.75 |
| Source plate | 6-well | NX Tilt | Flush with fresh medium | X | Span-8 pipette | 0.85 |
| Source plate | 6-well | NX Tilt | 1 mix and Transfer from well to 96-DW (well A1) | X | Span-8 pipette | 0.85 |
| Source plate | 6-well | NX Tilt | 1 mix and Transfer from well to 96-DW (well A1) | X | Span-8 pipette | 0.25 |
| Collection plate | 96-DW | Centrifuge | Centrifugation |  | Centrifuge 900 rpm/ 3 min |  |
| Collection plate | 96-DW | NX | Emptying supernatant to Wash Station |  | Span-8 pipette | 1.85 |
| Collection plate | 96-DW | NX | Add fresh medium (well A1) |  | Span-8 pipette | 1.7 |
| Collection plate | 96-DW | NX | Add fresh medium (wells B1 to H1) |  | Span-8 pipette | 1.295 |
| Collection plate | 96-DW | NX | 4 mixes A1 and Splitting well A1 into wells B1 to H1 |  | Span-8 pipette | 0.2285 |
| Destination plates | 6-well | NX Tilt | Emptying Matrigel from 7 wells | X | Span-8 pipette | 1.8 |
| Collection plate | 96-DW | NX | 4 Mixes and Transfer to 6 destination wells |  | Span-8 pipette | 1.5 |
| Collection plate | 96-DW | NX | Transfer to 7^th^ destination well |  | Span-8 pipette | 1.5 |
| Destination plates | 6-well | Cytomat 37 °C | Incubation |  | Cytomat 37 °C |  |

6-well = 6-well plate, 96-DW = 96-deepwell plate, NX = Biomek NXp, NX Tilt = Amplius (AIG) 3D Tilting device, FX = Biomek FX

**Supplementary Table 3: smNPC passaging from one 6-well to seven 6-wells**

| **Name** | **Labware** | **Position** | **Action** | **Plate tilted** | **Device** | **Volume in ml** |
| --- | --- | --- | --- | --- | --- | --- |
| Source plate | 6-well | NX Tilt | Emptying well to wash station | X | Span-8 pipette | 1.5 |
| Source plate | 6-well | NX Tilt | Add accutase |  | Span-8 pipette | 1 |
| Source plate | 6-well | Cytomat 37 °C | Incubation 10 min |  | Cytomat 37 °C |  |
| Source plate | 6-well | FX BioShake | Shaking 2000 rpm / 1 min |  | BioShake 3000 |  |
| Source plate | 6-well | NX Tilt | 8 Mixes and transfer to 96-DW in A1 | X | Span-8 pipette | 0.75 |
| Source plate | 6-well | NX Tilt | Flush with fresh medium | X | Span-8 pipette | 0.85 |
| Source plate | 6-well | NX Tilt | 8 Mixes and transfer to 96-DW in A1 | X | Span-8 pipette | 0.85 |
| Collection plate | 96-DW | Centrifuge | Centrifuge 900 rpm/ 3 min |  | Centrifuge |  |
| Collection plate | 96-DW | NX | Emptying supernatant from A1 to wash station |  | Span-8 pipette | 1.5 |
| Collection plate | 96-DW | NX | Add fresh medium to A1 and 5 mixes |  | Span-8 pipette | 1.2 |
| Collection plate | 96-DW | Centrifuge | Centrifuge 900 rpm/ 3 min |  | Centrifuge |  |
| Collection plate | 96-DW | NX | Emptying supernatant from A1 to wash station |  | Span-8 pipette | 1.2 |
| Collection plate | 96-DW | NX | Add fresh medium in A1 and 5 mixes |  | Span-8 pipette | 0.85 |
| Collection plate | 96-DW | NX | 12 Mixes 800ul in 96-DW in A1 |  | Span-8 pipette | 0.8 |
| Collection plate | 96-DW | NX | Split in 7 wells B1 to H1 |  | Span-8 pipette | 7x200 |
| Collection plate | 96-DW | NX | Add fresh medium to B1 to H1 |  | Span-8 pipette | 1.35 |
| Destination plate 1 | 6-well | NX Tilt | Emptying matrigel to wash station | X | Span-8 pipette | 1.5 |
| Destination plate 2 | 6-well | NX Tilt | Emptying matrigel to wash station | X | Span-8 pipette | 1.5 |
| Collection plate | 96-DW | NX | 12 Mixes and Transfer well B1 to destination well |  | Span-8 pipette | 1.5 |
| Collection plate | 96-DW | NX | 12 Mixes and Transfer C1 to H1 to destination wells |  | Span-8 pipette | 1.5 |
| Destination plates | 6-well | Cytomat 37 °C | Incubation |  | Cytomat 37 °C |  |

6-well = 6-well plate, 96-DW = 96-deepwell plate, NX = Biomek NXp, NX Tilt = Amplius (AIG) 3D Tilting device

**Supplementary Table 4: Fibroblast passaging from three 6-wells to one 1-well plate**

| **Name** | **Labware** | **Position** | **Action** | **Tilted plate** | **Device** | **Volume in ml** |
| --- | --- | --- | --- | --- | --- | --- |
| Source plate | 6-well | NX Tilt | Emptying to wash station | X | Span-8 pipette | 1.5 |
| Source plate | 6-well | NX Tilt | Add PBS |  | Span-8 pipette | 1.5 |
| Source plate | 6-well | NX Tilt | Emptying to wash station | X | Span-8 pipette | 1.5 |
| Source plate | 6-well | NX Tilt | Add Trypsin 0.25% |  | Span-8 pipette | 0.5 |
| Source plate | 6-well | Cytomat 37 °C | Incubation 5 min |  | Cytomat 37 °C |  |
| Source plate | 6-well | FX BioShake | Shaking 1500 rpm / 1 min |  | BioShake 3000 |  |
| Source plate | 6-well | NX Tilt | 8 Mixes and Transfer to 96-DW (to C1 to E1) | X | Span-8 pipette | 0.5 |
| Source plate | 6-well | NX Tilt | Flush with fresh medium | X | Span-8 pipette | 1.5 |
| Source plate | 6-well | NX Tilt | 8 Mixes and Transfer to 96-DW (to C1 to E1) | X | Span-8 pipette | 1.5 |
| Collection plate | 96-DW | NX Tilt | 12 Mixes 800ul in C1 to E1 |  | Span-8 pipette | 0.8 |
| Collection plate | 96-DW | NX | Split C1 to E1 into A1 to H1 (8wells) |  | Span-8 pipette | 8x750 |
| Collection plate | 96-DW | NX | Add fresh medium A1 to H1 |  | Span-8 pipette | 8x750 |
| Collection plate | 96-DW | NX | 10 Mixes |  | Span-8 pipette | 0.8 |
| Destination plate | 1-well | NX Tilt | Emptying water to wash station | X | Span-8 pipette | 12 |
| Collection plate | 96-DW | NX | 4 Mixes and Transfer to destination plate |  | Span-8 pipette | 12 |
| Destination plates | 1-well | FX BioShake | Shaking 1000 rpm / 1min |  | Bioshake 3000 |  |
| Destination plates | 1-well | Cytomat 37 °C | Incubation |  | Cytomat 37 °C |  |

6-well = 6-well plate, 96-DW = 96-deepwell plate, 1-well = Omnitray 1-well plate, NX = Biomek NXp, NX Tilt = Amplius (AIG) 3D Tilting device

**Supplementary Table 5: Fibroblast passaging from one 1-well plate to three 1-well plates**

| **Name** | **Labware** | **Position** | **Action** | **Tilted plate** | **Device** | **Volume in ml** |
| --- | --- | --- | --- | --- | --- | --- |
| Source plate | 1-well | NX Tilt | Emptying 1 well plates | X | Span-8 pipette | 12 |
| Source plate | 1-well | NX Tilt | Add PBS |  | Span-8 pipette | 12 |
| Source plate | 1-well | NX Tilt | Emptying | X | Span-8 pipette | 12 |
| Source plate | 1-well | NX Tilt | Add Trypsin 0.25% |  | Span-8 pipette | 5 |
| Source plate | 1-well | Cytomat 37 °C | Incubation 5 min |  | Cytomat 37 °C |  |
| Source plate | 1-well | FX BioShake | Shaking 2000 rpm / 1min |  | Bioshake 3000 |  |
| Source plate | 1-well | NX Tilt | 8 Mixes and Transfer to Reservoir | X | Span-8 pipette | 5 |
| Source plate | 1-well | NX Tilt | Flush with fresh medium | X | Span-8 pipette | 10 |
| Collection plate | 300 mL Reservoir | NX Tilt | 8 Mixes and Transfer to Reservoir containing 36 mL medium | X | Span-8 pipette | 10 |
| Collection plate | 300 mL Reservoir | FX | 10 Mixes in reservoir |  | 96-head pipette | 15 |
| Destination plates | 1-well | FX | Emptying water to wash station |  | 96-head pipette | 12 |
| Reservoir | 300 mL Reservoir | FX | 10 Mixes and transfer in destination plates |  | 96-head pipette | 12 |
| Destination plates | 1-well | FX BioShake | Shaking 1000 rpm / 1min |  | Bioshake 3000 |  |
| Destination plates | 1-well | Cytomat 37 °C | Incubation of 6 plates |  | Cytomat 37 °C |  |

1-well = Omnitray 1-well plate, NX = Biomek NXp, NX Tilt = Amplius (AIG) 3D Tilting device, FX = Biomek FX

**Supplementary Table 6: iPSC passaging from two 6-wells to one 1-well plate**

| **Name** | **Labware** | **Position** | **Action** | **Plate tilted** | **Device** | **Volume in ml** |
| --- | --- | --- | --- | --- | --- | --- |
| Source plate | 6-well | NX Tilt | Emptying to wash station | X | Span-8 pipette | 1.5 |
| Source plate | 6-well | NX Tilt | Add PBS in 6w |  | Span-8 pipette | 0.85 |
| Source plate | 6-well | NX Tilt | Empty PBS to wash station | X | Span-8 pipette | 0.85 |
| Source plate | 6-well | NX Tilt | Add PBS in 6w |  | Span-8 pipette | 0.85 |
| Source plate | 6-well | NX Tilt | Empty PBS to wash station | X | Span-8 pipette | 0.85 |
| Source plate | 6-well | NX Tilt | Add PBS in 6w |  | Span-8 pipette | 0.85 |
| Source plate | 6-well | NX Tilt | Empty PBS to wash station | X | Span-8 pipette | 0.85 |
| Source plate | 6-well | NX Tilt | Add EDTA in 6w |  | Span-8 pipette | 1 |
| Source plate | 6-well | Cytomat 37 °C | Incubation 6 min |  | Cytomat 37 °C |  |
| Source plate | 6-well | FX BioShake | Shaking 2000 rpm / 1 min |  | BioShake 3000 |  |
| Source plate | 6-well | NX Tilt | Flush cells | X | Span-8 pipette |  |
| Source plate | 6-well | NX Tilt | 1 mix and Transfer 6w to 96-DW (A1 and B1) | X | tilter | 0.75 |
| Source plate | 6-well | NX Tilt | Flush with fresh medium | X | Span-8 pipette | 0.85 |
| Source plate | 6-well | NX Tilt | 1 mix and transfer to 96-DW (A1 and B1) | X | Span-8 pipette | 0.85 |
| Source plate | 6-well | NX Tilt | Transfer to 96-DW (A1 and B1) | X | Span-8 pipette | 0.25 |
| Collection plate | 96-DW | Centrifuge | Centrifugation |  | Centrifuge 900 rpm/ 3 min |  |
| Collection plate | 96-DW | NX | Emptying supernatant to wash station |  | Span-8 pipette | 1.8 |
| Collection plate | 96-DW | NX | Add fresh medium |  | Span-8 pipette | 1.85 |
| Collection plate | 96-DW | NX | A1 to B1 : 4 mixes and transfer to C1 to F1 |  | Span-8 pipette | 0.6 |
| Collection plate | 96-DW | NX | Add fresh medium to A1 to F1 |  | Span-8 pipette | 0.6 |
| Destination plate | 1-well | NX Tilt | Emptying matrigel to wash station | X | Span-8 pipette | 12 |
| Medium | 300 mL Reservoir | NX | Transfer to destination plate |  | Span-8 pipette | 5.1 |
| Collection plate | 96-DW | NX | 4 mixes and transfer A1 to F1 of DW to 1 destination plate |  | Span-8 pipette | 7.2 |
| Destination plate | 1-well | Cytomat 37 °C | Incubation |  | Cytomat  37 °C |  |

6-well = 6-well plate, 96-DW = 96-deepwell plate, 1-well = Omnitray 1-well plate, NX = Biomek NXp, NX Tilt = Amplius (AIG) 3D Tilting device

**Supplementary Table 7: iPSC passaging from one 1-well plate to five 1-well plates**

| **Name** | **Labware** | **Position** | **Action** | **Tilted plate** | **Device** | **Volume in ml** |
| --- | --- | --- | --- | --- | --- | --- |
| Source plate | 1-well | NX Tilt | Emptying well to wash station | X | Span-8 pipette | 12 |
| Source plate | 1-well | NX Tilt | Add PBS in 1w |  | Span-8 pipette | 5 |
| Source plate | 1-well | NX Tilt | Empty PBS to wash station | X | Span-8 pipette | 5 |
| Source plate | 1-well | NX Tilt | Add PBS in 1w |  | Span-8 pipette | 5 |
| Source plate | 1-well | NX Tilt | Empty PBS to wash station | X | Span-8 pipette | 5 |
| Source plate | 1-well | NX Tilt | Add PBS in 1w |  | Span-8 pipette | 5 |
| Source plate | 1-well | NX Tilt | Empty PBS towash station | X | Span-8 pipette | 5 |
| Source plate | 1-well | NX Tilt | Add EDTA in 1w |  | Span-8 pipette | 7 |
| Source plate | 1-well | Cytomat 37 °C | Incubation 15 min |  | Cytomat 37 °C |  |
| Source plate | 1-well | FX BioShake | Shaking 2000 rpm / 1 min |  | BioShake 3000 |  |
| Source plate | 1-well | NX Tilt | Transfer into A1 to H1 96-DW | X | Span-8 pipette | 0.875 |
| Source plate | 1-well | NX Tilt | Flush with fresh medium | X | Span-8 pipette | 5 |
| Source plate | 1-well | NX Tilt | Transfer into A1 to H1 96-DW | X | Span-8 pipette | 0.625 |
| Source plate | 1-well | NX Tilt | Flush with fresh medium | X | Span-8 pipette | 4 |
| Source plate | 1-well | NX Tilt | Transfer into A1 to H1 96-DW | X | Span-8 pipette | 0.5 |
| Collection plate | 96-DW | Centrifuge | Centrifugation |  | Centrifuge 900 rpm/ 3 min |  |
| Collection plate | 96-DW | NX | Emptying supernatant from A1 to H1 to wash station |  | Span-8 pipette | 1.875 |
| Collection plate | 96-DW | NX | Add fresh medium in A1 to H1 |  | Span-8 pipette | 1.8 |
| Destination plates | 1-well | FX | Emptying Matrigel for 5 plates to wash station |  | 96 pipette head | 12 |
| Medium | 300 mL Reservoir | FX | Transfer medium to destination plates |  | 96 pipette head | 7.68 |
| Collection plate | 96-DW | NX | Mix 2 times A1 to H1 well to transfer |  | Span-8 pipette | 2.8 |
| Collection plate | 96-DW | NX | Transfer 5 times A1 to H1 in 5 1w plates |  | Span-8 pipette | 3.04 |
| Destination plates | 1-well | Cytomat 37 °C | Incubation of 5 plates |  | Cytomat 37 °C |  |

6-well = 6-well plate, 96-DW = 96-deepwell plate, 1-well = Omnitray 1-well plate, NX = Biomek NXp, NX Tilt = Amplius (AIG) 3D Tilting device, FX = Biomek FX

**Supplementary Table 8: smNPC passaging from one 6-well to one 1-well plate**

| **Name** | **Labware** | **Position** | **Action** | **Plate tilted** | **Device** | **Volume in ml** |
| --- | --- | --- | --- | --- | --- | --- |
| Source plate | 6-well | NX Tilt | Emptying well to wash station | X | Span-8 pipette | 1.5 |
| Source plate | 6-well | NX Tilt | Add accutase |  | Span-8 pipette | 1 |
| Source plate | 6-well | Cytomat 37 °C | Incubation 10 min |  | Cytomat 37 °C |  |
| Source plate | 6-well | FX BioShake | Shaking 2000 rpm / 1 min |  | BioShake 3000 |  |
| Source plate | 6-well | NX Tilt | Flush cells | X | Span-8 pipette |  |
| Source plate | 6-well | NX Tilt | 8 Mixes and transfer to A1 of 96-DW | X | Span-8 pipette | 0.75 |
| Source plate | 6-well | NX Tilt | Flush with fresh medium | X | Span-8 pipette | 0.85 |
| Source plate | 6-well | NX Tilt | 8 Mixes and transfer to A1 of96-DW | X | Span-8 pipette | 1.1 |
| Collection plate | 96-DW | Centrifuge | Centrifugation 900 rpm/ 3 min |  | Centrifuge |  |
| Collection plate | 96-DW | NX | Emptying supernatant of A1 to wash station |  | Span-8 pipette | 1.825 |
| Collection plate | 96-DW | NX | Add fresh medium and 5 mixes to A1 |  | Span-8 pipette | 1.85 |
| Collection plate | 96-DW | NX | Add Medium in B1 to H1 |  | Span-8 pipette | 0.675 |
| Collection plate | 96-DW | NX | 4 Mixes and transfer in B1 to H1 |  | Span-8 pipette | 0.225 |
| Collection plate | 96-DW | NX | Medium in A1 to H6 |  | Span-8 pipette | 0.675 |
| Destination plates | 1-well | NX Tilt | Emptying matrigel to wash station | X | Splan8 pipette | 12 |
| Medium | 300 mL Reservoir | NX | Transfer medium to destination plates |  | Splan8 pipette | 4,8 |
| Collection plate | 96-DW | NX | 4 Mixes and Transfer DW wells by column in each 1w plate |  | Span-8 pipette | 7,2 |
| Destination plates | 1-well | Cytomat 37 °C | Incubation |  | Cytomat 37 °C |  |

6-well = 6-well plate, 96-DW = 96-deepwell plate, 1-well = Omnitray 1-well plate, NX = Biomek NXp, NX Tilt = Amplius (AIG) 3D Tilting device

**Supplementary Table 9: smNPC passaging from one 1-well plate to six 1-well plates**

| **Name** | **Labware** | **Position** | **Action** | **Tilted plate** | **Device** | **Volume in ml** |
| --- | --- | --- | --- | --- | --- | --- |
| Source plate | 1-well | NX Tilt | Emptying well to wash station | X | Span-8 pipette | 12 |
| Source plate | 1-well | NX Tilt | Add accutase |  | Span-8 pipette | 8 |
| Source plate | 1-well | Cytomat 37 °C | Incubation |  | Cytomat 37 °C |  |
| Source plate | 1-well | FX | Shaking 2000 rpm / 1 min |  | BioShake 3000 |  |
| Source plate | 1-well | NX Tilt | 8 Mixes and transfer to 8 wells of 96-DW | X | Span-8 pipette | 1 |
| Source plate | 1-well | NX Tilt | Flush with fresh medium | X | Span-8 pipette | 5 |
| Source plate | 1-well | NX Tilt | 8 Mixes and transfer to 8 wells of 96-DW | X | Span-8 pipette | 0.625 |
| Collection plate | 96-DW | Centrifuge | Centrifugation 900 rpm/ 3 min |  | Centrifuge |  |
| Collection plate | 96-DW | NX | Emptying supernatant to wash station |  | Span-8 pipette | 1.48 |
| Collection plate | 96-DW | NX | Add fresh medium |  | Span-8 pipette | 1.5 |
| Collection plate | 96-DW | NX | 12 Mixes and Transfer to reservoir containing 60 mL |  | Span-8 pipette | 1.65 |
| Reservoir | 300 mL Reservoir | FX | 10 Mixes using 96 tips |  | 96-head pipette | 16.32 |
| Destination plates | 1-well | FX | Emptying matrigel to wash station |  | 96-head pipette | 12 |
| Collection plate | 96-DW | FX | Transfer to 6 Destination plates |  | 96-head pipette | 12 |
| Destination plates | 1-well | Bioshake 3000 | Shaking 1000 rpm / 1 min |  | BioShake 3000 |  |
| Destination plates | 1-well | Cytomat 37 °C | Incubation of 6 plates |  | Cytomat 37 °C |  |

96-DW = 96-deepwell plate, 1-well = Omnitray 1-well plate, NX = Biomek NXp, NX Tilt = Amplius (AIG) 3D Tilting device, FX = Biomek FX

**Supplementary Table 10: Protocol durations**

| **Protocol** | **Protocol for one cell line** | **Duration in minutes** | **Optimization by combining different lines on one plate** | **Optimized duration in minutes** |
| --- | --- | --- | --- | --- |
| Fibroblast maintenance splitting | Splitting one 6-well into three 6-wells | 53 | Splitting an entire 6-well plate into three 6-well plates | 127 |
| Fibroblast expansion 1^st^ step | Splitting three 6-wells into one 1-well plate | 94 | Splitting an entire 6-well plate into two 1-well plates | 123 |
| Fibroblast expansion 2^nd^ step | Splitting one 1-well plate into three 1-well plates | 67 | - | - |
| iPSC maintenance splitting | Splitting one 6-well into seven 6-wells | 70 | Splitting an entire 6-well plate into seven 6-well plates | 210 |
| iPSC expansion 1^st^ step | Splitting two 6-wells into one 1-well plate | 68 | Splitting an entire 6-well plate into three 1-well plates | 106 |
| iPSC expansion 2^nd^ step | Splitting one 1-well plate into five 1-well plates | 102 | - | - |
| smNPC maintenance splitting | Splitting one 6-well into seven 6-wells | 80 | Splitting an entire 6-well plate into seven 6-well plates | 186 |
| smNPC expansion 1^st^ step | Splitting one 6-well into one 1-well plate | 67 | Splitting an entire 6-well plate into six 1-well plates | 191 |
| smNPC expansion 2^nd^ step | Splitting one 1-well plate into six 1-well plates | 105 | - | - |
